# Pre-treatment lactate dehydrogenase levels as predictor of efficacy of first-line bevacizumab-based therapy in metastatic colorectal cancer patients

**DOI:** 10.1038/bjc.2012.17

**Published:** 2012-02-07

**Authors:** M Scartozzi, R Giampieri, E Maccaroni, M Del Prete, L Faloppi, M Bianconi, E Galizia, C Loretelli, L Belvederesi, A Bittoni, S Cascinu

**Affiliations:** 1 Clinica di Oncologia Medica, AO Ospedali Riuniti, Università Politecnica delle Marche, Via Conca, Ancona, Italy; 2 Scuola di Specializzazione in Oncologia Medica, Università Politecnica delle Marche, Ancona, Italy; 3 Oncologia Medica, Ospedale Profili, Fabriano, Italy

**Keywords:** bevacizumab, colorectal cancer, LDH, predictive factors

## Abstract

**Background:**

Lactate dehydrogenase (LDH) represents a predictive factor in colorectal cancer patients treated with the angiogenesis inhibitor PTK/ZK. We explored the role of pre-treatment LDH serum levels in colorectal cancer patients receiving first-line bevacizumab.

**Methods:**

Metastatic colorectal cancer treated with first-line bevacizumab was eligible. A control group including all consecutive patients treated with chemotherapy alone was also considered. Pre-treatment LDH serum levels were collected for all cases.

**Results:**

Median progression-free survival (PFS) in the control group for patients with high and low LDH levels was 4.2 and 8 months, respectively (*P*=0.0003). Median overall survival (OS) was 19.6 and 34.9 months for patients with high and low LDH levels, respectively (*P*=0.0014). In the bevacizumab group, partial responses were seen in 14 (58%) high-LDH and 8 (14%) low-LDH patients (*P*=0.0243), respectively, median PFS was 7.3 and 8.5 months, respectively (*P*=0.2), and median OS was 22 and 26.6 months, respectively (*P*=0.7).

**Conclusion:**

High LDH levels correlated with worse prognosis. Bevacizumab seemed capable of improving clinical outcome in this specific group of patients who usually present with an adverse natural history. The improved response rate also suggests a role for LDH as a predictive marker.

The VEGF-driven tumour pathway has been demonstrated to represent a novel therapeutic target for an innovative class of antineoplastic agents. Among these antiangiogenetic-targeted treatment modalities, the anti-VEGF monoclonal antibody bevacizumab has become a new standard of care for first-line treatment of metastatic colorectal cancer (Douillard *et al*, 2000; Hurwitz *et al*, 2004; Koehne *et al*, 2005).

The expanding role of anti-VEGF treatment for metastatic colorectal tumour patients, along with the growing number of cases potentially requiring such a treatment approach, made the need for a reliable identification of responding tumours increasingly crucial (Folkman, 1971).

However, if on the one hand clinical reports with the use of bevacizumab have shown promising results, on the other hand in these trials no predictive markers of response or resistance were identified.

The biological link between hypoxia, lactate dehydrogenase (LDH) levels and the tumour-driven angiogenesis pathway through the abnormal activation of the hypoxia-inducible factor 1-*α* (HIF1-*α*) is well established. Hypoxia-inducible factor 1-*α* is an important transcription factor that upregulates a series of genes involved in glycolytic metabolism, angiogenesis, cell survival and erythropoiesis. Among the others, HIF1-*α* also regulates transcription of several glycolytic enzymes, such as LDH (Maxwell *et al*, 2001). More specifically, it has been suggested that HIF1-*α* overexpression was linked to the LDH-5 isoform activity (Koukourakis *et al*, 2003).

In preclinical models, upregulation of LDH has been suggested to ensure both an efficient anaerobic/glycolytic metabolism and a reduced dependence on oxygen under hypoxic conditions in tumour cells.

As LDH and pro-angiogenesis factors are regulated by the same HIF1-*α*-driven molecular pathway, high LDH levels are concomitantly present along with abnormal activation of the VEGF pathway (Harris, 2002).

According to this biological assumption, Azuma *et al* (2007) demonstrated that high LDH serum levels were associated with tumour overexpression of VEGFA and VEGFR-1. As a clinical consequence, it has been speculated that LDH levels may represent an indirect indicator of activated tumour angiogenesis and ultimately of worse prognosis (Tas *et al*, 2001a, 2001b).

In colorectal cancer, LDH-5 overexpression has been demonstrated to significantly correlate with an increased risk of metastases, and high LDH serum levels have been implicated in determining a worse prognosis (Koukourakis *et al*, 2005; Wu *et al*, 2010). This finding has been confirmed in other tumour types as well (Brizel *et al*, 2001; Yuce *et al*, 2001).

The role of LDH in patients receiving antiangiogenic therapy is more controversial. Available evidences in this setting are limited to those deriving from the CONFIRM-1 and -2 trials. In these studies, PTK/ZK (vatalanib), an oral inhibitor of VEGF receptors, was used in combination with chemotherapy (FOLFOX) for, respectively, first- and second-line therapy of advanced colorectal cancer (Hecht *et al*, 2011; Van Cutsem *et al*, 2011). Both trials did not meet the primary end point. However, in an exploratory *post hoc* analysis, median progression-free survival (PFS) improved with the use of PTK/ZK in patients with high LDH serum levels, thus suggesting that LDH might be a predictive marker for antiangiogenic treatment.

Recently, Koukourakis *et al* (2011) also demonstrated that serum LDH and tissue LDH-5 are complementary features that may help characterising the activity of LDH in colorectal cancer.

On the other hand, data in colorectal cancer patients receiving first-line bevacizumab are lacking and could be relevant for treatment strategy and therapeutic decision in clinical practice. The aim of our study was to explore a possible link between pre-treatment LDH levels and clinical outcome in advanced colorectal cancer patients treated with first-line chemotherapy and bevacizumab.

## Patients and methods

### Patient selection

All patients with histologically proven metastatic colorectal cancer consecutively treated with a first-line chemotherapy doublet and bevacizumab at our Institution were eligible for our analysis. A historical control group was also created, including all consecutive histologically proven metastatic colorectal cancer patients treated at our Institution with a chemotherapy doublet before the introduction of bevacizumab in clinical practice. Pre-treatment LDH serum levels were collected for all patients.

The following first-line chemotherapy doublets were used: modified FOLFIRI (irinotecan 180 mg m^−2^ d1, 5FU bolus 400 mg m^−2^ d1, 5FU 2400 mg m^−2^ continuous infusion for 46 h every 2 weeks) or FOLFOX-6 (oxaliplatin 85 mg m^−2^ d1, 5FU bolus 400 mg m^−2^ d1, 5FU 2400 mg m^−2^ continuous infusion for 46 h, every 2 weeks) or XELOX (oxaliplatin 130 mg m^−2^ d1, capecitabine 2000 mg m^−2^ d1 to 14 every 3 weeks) either in combination with bevacizumab (5 mg kg^−1^ every 2 weeks or 7.5 mg kg^−1^ every 3 weeks) or without bevacizumab.

Tumour response was evaluated every 8 weeks by clinicians’ assessment and according to the Response Evaluation Criteria in Solid Tumors (RECIST).

### Statistical analysis

Statistical analysis was performed with the MedCalc package (MedCalc v.9.4.2.0, MedCalc Software bvba, Mariakerke, Belgium).

Receiver operating characteristics (ROC) curve analysis was performed to determine a cutoff value for pre-treatment LDH levels.

The association between categorical variables was analysed by *χ*^2^-test. Survival distribution was estimated by the Kaplan–Meier method. Significant differences in probability of relapsing between the strata were evaluated by log-rank test. Cox multiple regression analysis was used to assess the role of variables that resulted to be significant at univariate analysis.

Tested variables included gender (male *vs* female), age (<65 *vs* ⩾65 years), grade of tumour differentiation (well *vs* moderately differentiated and undifferentiated), Eastern Cooperative Oncology Group Performance Status Scale (ECOG PS) (<2 *vs* ⩾2) and LDH serum level (⩽588 *vs* >588 mg dl^−1^).

The heterogeneity of the effect of LDH levels between bevacizumab and historical control group was explored by using a statistical test for interaction, applied through a Cox model for PFS and overall survival (OS).

A significant level of 0.05 was chosen to assess the statistical significance.

For statistical analysis, OS and PFS were defined, respectively, as the interval between the start of chemotherapy to death or last follow-up visit, and as the interval between the start of chemotherapy to clinical progression or death, or last follow-up visit if not progressed.

## Results

**Figure 1:**
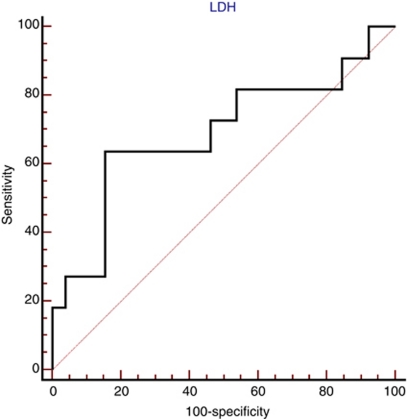
Receiver operating characteristics analysis based on pre-treatment LDH serum levels results with OS as end point. In this model, sensitivity was 63.64% (95% CI: 40.7–82.8) and specificity was 84.62% (95% CI: 71.9–93.1). Area under the curve was 0.689, *P*=0.0077.

Globally, 220 patients with advanced colorectal cancer receiving first-line chemotherapy were available for our analysis. In all, 82 patients were treated with a chemotherapy doublet (either oxaliplatin or irinotecan in combination with fluoropyrimidines) in combination with bevacizumab (bevacizumab group; accrual interval 2005–2011), whereas the remaining 138 patients received chemotherapy (either oxaliplatin or irinotecan in combination with fluoropyrimidines) alone (historical control group; accrual interval 1999–2005). The two groups of patients were comparable for all major clinical characteristics such as age at diagnosis, sex, metachronous *vs* synchronous metastatic involvement, previous adjuvant chemotherapy, number of metastatic sites and proportion receiving second-line treatment (Table 1). The cutoff point with the highest sensitivity and specificity for estimating pre-treatment LDH serum levels as a function of treatment of clinical activity was set at ⩽588 mg dl^-1^ after ROC curve analysis (Figure 1). Consequently, patients showing a pre-treatment LDH serum level ⩾588 mg dl^−1^ were classified as high-LDH patients, in contrast to patients with pre-treatment LDH serum level lower than 588 mg dl^−1^ (low-LDH patients). Globally, 44 patients (20%) showed high pre-treatment LDH levels.

### Role of pre-treatment LDH levels in the historical control group

In all, 138 patients were available for analysis in this group. Of these, 49 patients showed a partial response (35%), 1 patient obtained a complete remission (1%), 32 patients achieved a stable disease (23%) and 40 patients (29%) progressed during chemotherapy. The remaining 16 patients (12%) were not assessable for response. In the chemotherapy-alone group, 20 patients (15%) had high pre-treatment LDH levels. The remaining 118 patients (85%) presented with low pre-treatment LDH levels. The two subgroups of patients were comparable for all major clinical characteristics (Table 1).

In high- and low-LDH patients, we observed a partial response in 4 (20%) and 46 (39%) patients, respectively (*P*=0.1671), whereas progressive disease was observed in 14 (70%) and 26 (22%) patients with, respectively, high and low pre-treatment LDHlevels (*P*<0.0001; Table 2).

**Figure 2:**
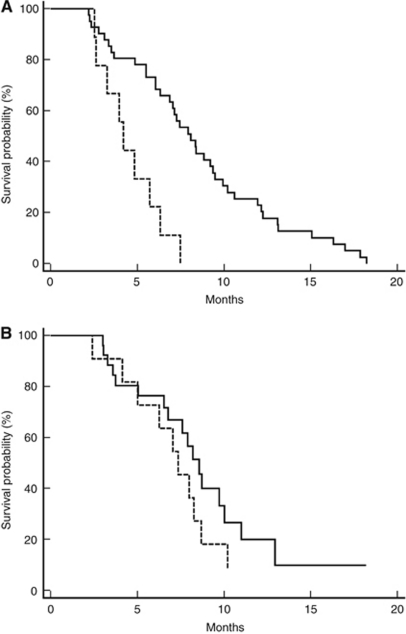
(**A**) Kaplan–Meier curves for median PFS of colorectal cancer patients in the historical control group showing high pre-treatment LDH serum levels (- - - - - - -) and low pre-treatment serum levels (———) (4.2 *vs* 8 months, *P*=0.0003). (**B**) Kaplan–Meier curves for median PFS of colorectal cancer patients in the bevacizumab group showing high pre-treatment LDH serum levels (- - - - - - -) and low pre-treatment serum levels (———) (8.5 *vs* 7.3 months, *P*=0.2).

**Figure 3:**
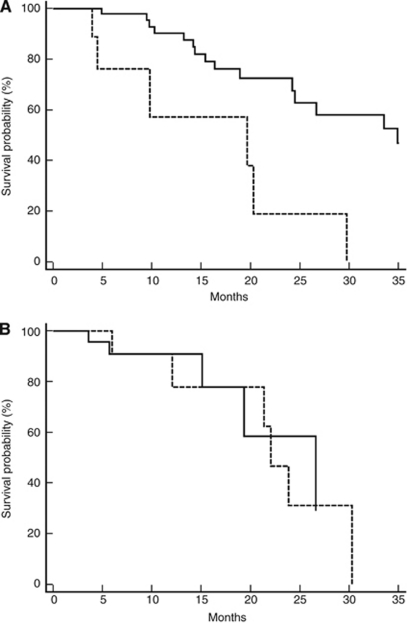
(**A**) Kaplan–Meier curves for median OS of colorectal cancer patients in the historical control group showing high pre-treatment LDH serum levels (- - - - - - -) and low pre-treatment serum levels (———) (19.6 *vs* 34.9 months, *P*=0.0014). (**B**) Kaplan–Meier curves for median OS of colorectal cancer patients in the bevacizumab group showing high pre-treatment LDH serum levels (- - - - - - -) and low pre-treatment serum levels (———) (26.6 *vs* 22 months, *P*=0.7).

Median PFS and OS for patients in the historical control group were, respectively, 7.2 and 29.7 months. In this group, pre-treatment LDH levels were statistically related to both worse median PFS and OS. In particular, patients with high LDH levels achieved a median PFS of 4.2 months, whereas median PFS was 8 months for patients with low pre-treatment LDH levels (*P*=0.0003; HR: 0.2973; 95% CI: 0.0318–0.3543; Figure 2 and Table 2). Median OS was 19.6 and 34.9 months for patients with high and low pre-treatment LDH levels, respectively (*P*=0.0014; HR: 0.2484; 95% CI: 0.0188–0.3884; Figure 3 and Table 2). Among the other tested clinical parameters, median OS improved in male patients (median OS in male *vs* female patients: 33.5 *vs* 17.8 months, respectively, *P*=0.007) and in patients with a well-differentiated tumour (median OS in patients with well-differentiated *vs* moderately differentiated and undifferentiated tumours: not reached *vs* 25.5 months, respectively, *P*=0.007). At multivariate analysis, only LDH serum level maintained an independent prognostic value.

### Role of pre-treatment LDH levels in the bevacizumab group

In all, 82 patients were available for analysis in this group. Of these, 22 patients showed a partial response (27%), 40 patients obtained a stable disease (49%), whereas 12 patients (15%) progressed during treatment. In the remaining eight patients (9%), response was not assessable. No complete remissions were observed. In the bevacizumab group, 24 patients (29%) had high pre-treatment LDH levels. The remaining 58 patients (71%) presented with low pre-treatment LDH levels. The two subgroups of patients were comparable for all major clinical characteristics (Table 1). In high- and low-LDH patients, we observed a partial response in 14 (58%) and 8 (14%) patients, respectively (*P*=0.0243), whereas progressive disease was observed in 2 (8%) and 10 (17%) patients with, respectively, high and low pre-treatment LDH levels, respectively (*P*=0.48; Table 2). Median PFS was 8.5 and 7.3 months, respectively, for high- and low-LDH patients (*P*=0.2; HR: 0.6360; 95% CI: 0.2528–1.4185) (Figure 2 and Table 2). Median OS survival was 26.6 and 22 months, respectively, for patients showing high or low pre-treatment LDH levels (*P*=0.7; HR: 0.8480; 95% CI: 0.2307–2.9563) (Figure 3 and Table 2). All the other tested variables (age, gender, ECOG PS and tumour grade) failed to show a significant correlation with survival parameters.

### Combined results

**Figure 4:**
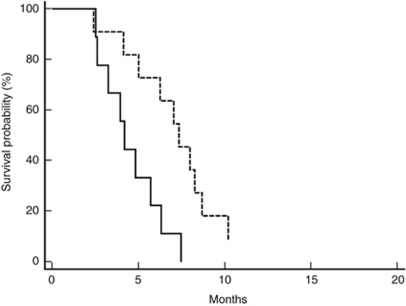
Kaplan–Meier curves for median PFS of colorectal cancer patients showing high pre-treatment LDH serum levels. Bevacizumab group (- - - - - - -) and historical control group (———) (8.5 *vs* 4.2 months, *P*=0.006).

We compared results from the bevacizumab and control groups according to pre-treatment LDH levels. No statistically significant differences were noticed for median OS. In the high-LDH group, response rate was higher in patients treated with bevacizumab (58% *vs* 20%, *P*=0.01). Accordingly, median PFS in the high-LDH group was in favour of patients treated with bevacizumab (*P*=0.006; HR: 3.6257; 95% CI: 1.5776–16.0715; Figure 4). The interaction test between LDH levels and treatment effect, in the bevacizumab group and historical control group, suggested that the relation of LDH levels with improved outcome was significantly associated with the effect of bevacizumab in terms of PFS (*P*=0.02), but not in terms of OS (*P*=0.07).

## Discussion

The role of LDH as a marker of clinical outcome in advanced colorectal cancer patients has been suggested in different clinical series. Previous reports in colorectal cancer patients indicated, in fact, that LDH upregulation was associated with an increased risk of both nodal and distant metastases (Tas *et al*, 2001a), and that high LDH serum levels could predict a decreased median OS and have a prognostic impact (De Gramont *et al*, 2000; Tas *et al*, 2001b; Tournigand *et al*, 2004, 2011). This analysis suggested that pre-treatment LDH levels could be considered a relevant factor for risk evaluation in colorectal cancer patients receiving chemotherapy. Besides representing a possible indicator of prognosis, LDH may also have a role in patient stratification for clinical trials investigating first-line therapy in colorectal tumours. However, although a biological relationship between LDH levels, tumour hypoxia and angiogenesis has been indicated in pre-clinical and clinical studies, the role of pre-treatment LDH levels in patients receiving anti-VEGF therapy remains substantially unexplored. The only available data about the role of LDH levels and an antiangiogenic treatment are those deriving from the recently published trials investigating PTK/ZK (vatalanib), an oral inhibitor of VEGF receptors, in first- and second-line therapy of advanced colorectal cancer. Both trials did not reach their primary end point, but in an exploratory *post hoc* analysis median PFS improved with the use of PTK/ZK in patients with high serum LDH level. No meaningful effect was seen on either response rate or OS. The strong association between LDH-5 expression and the activated VEGF pathway demonstrated in a study by Koukourakis *et al* (2006) may represent a biological rationale to explain anti-VEGF activity in the presence of high LDH levels.

Interestingly, our analysis suggested that metastatic colorectal cancer patients treated with bevacizumab and showing high pre-treatment LDH levels experienced an improved probability of response and an equivalent median PFS and OS when compared with patients presenting with low LDH levels. These findings may be considered in accordance with the observations derived from the CONFIRM-1 and -2, thus confirming an improved clinical outcome for patients with high LDH levels when treated with an anti-VEGF therapy. However, in contrast to the PTK/ZK results, we evidenced an improved response rate in the high-LDH group treated with bevacizumab (which is not present in neither the Hecht nor the Van Cutsem study; Hecht *et al*, 2011; Van Cutsem *et al*, 2011), whereas no advantages could be seen in our series with regard to median PFS. Many factors may have contributed to these apparently different profiles of activity. The different antiangiogenic treatment used (an anti-VEGF monoclonal antibody in our case *vs* an oral VEGF receptor tyrosine kinase inhibitor in the CONFIRM trials) should be considered relevant. Although we have limited data on the activity profile of PTK/ZK, we can in fact assume that vatalanib may be more effective in improving PFS than response rate in the high-LDH subgroup of patients, whereas bevacizumab demonstrated also an impact in determining response rate across trials. We also know that radiological evaluation of response could represent a confounding factor for patients receiving antiangiogenic treatment (Chun *et al*, 2009).

In our experience, treatment with bevacizumab seemed to be capable of improving clinical outcome in a specific group of patients who usually present with an adverse natural history. In this group of patients, bevacizumab seemed to act as a clinical outcome ‘equaliser’ inducing a reversal of a poor prognosis. The finding of an improved response rate and median PFS for patients with high pre-treatment LDH levels receiving bevacizumab over patients with the same LDH levels not receiving bevacizumab could represent a corroboration to this observation.

The interaction test between LDH levels and treatment effect, in the bevacizumab group and historical control group, in fact suggested that the improved outcome in high-LDH patients was significantly associated with the effect of bevacizumab on PFS. Our results, if confirmed in a larger data set, may have relevant implications for the choice of a first-line treatment for advanced colorectal cancer patients. We can in fact speculate that, in colorectal cancer patients with high LDH levels, the use of an antiangiogenic treatment in combination with chemotherapy may improve clinical outcome and allow a better management of the metastatic disease.

## Figures and Tables

**Table 1:** Patients characteristics

	***N***	**Median age (range)**	**Males (%)**	**Metachronous disease (%)**	**Prior adjuvant chemotherapy (%)**	**Metastatic involvement (A–B–C–D** **)**	**2nd line (%)**
Bevacizumab group	82	61 (29–75)	52 (64%)	52 (64%)	40 (48%)	40 (48%) A	61 (75%)
						10 (12%) B	
						18 (22%) C	
						14 (18%) D	
							
Control group	138	63 (29–77)	91 (66%)	90 (65%)	62 (44%)	63 (45%) A	103 (75%)
						18 (14%) B	
						30 (22%) C	
						27 (19%) D	
							
All	220	61 (29–77)	153 (65%)	142 (64%)	102 (46%)	103 (47%) A	164 (75%)
						28 (13%) B	
						48 (22%) C	
						41 (18%) D	

Abbreviations: A=liver involvement only; B=lung involvement only; C=liver+lung involvement; D=other metastatic sites.

**Table 2:** Response rate according to pre-treatment LDH levels in the bevacizumab group and in the control group

	**Control group**			**Bevacizumab group**		
	**LDH <588**	**LDH >588**	***P*-value**	**LDH <558**	**LDH >588**	***P*-value**
Partial+complete response	46 (39%)	4 (20%)	0.1671	8 (14%)	14 (58%)	0.0243
Stable disease	46 (39%)	2 (10%)	0.0237	40 (69%)	8 (36%)	0.0063
Progressive disease	26 (22%)	14 (70%)	<0.0001	10 (17%)	2 (8%)	0.48
	118	20		58	24	
	138			82		

Abbreviation: LDH=lactate dehydrogenase.
